# Recent Clinical Trials in Osteoporosis: A Firm Foundation or Falling Short?

**DOI:** 10.1371/journal.pone.0156068

**Published:** 2016-05-18

**Authors:** Karen Barnard, Wanda C. Lakey, Bryan C. Batch, Karen Chiswell, Asba Tasneem, Jennifer B. Green

**Affiliations:** 1 Department of Medicine, Duke University School of Medicine, Durham, North Carolina, United States of America; 2 Department of Medicine, Durham Veterans Affairs Medical Center, Durham, North Carolina, United States of America; 3 Duke Clinical Research Institute, Duke University School of Medicine, Durham, North Carolina, United States of America; 4 Office of Research Informatics, Duke University School of Medicine, Durham, North Carolina, United States of America; Klinikum rechts der Isar - Technical University Munich - TUM, GERMANY

## Abstract

The global burden of osteoporotic fractures is associated with significant morbidity, mortality, and healthcare costs. We examined the ClinicalTrials.gov database to determine whether recently registered clinical trials addressed prevention and treatment in those at high risk for fracture. A dataset of 96,346 trials registered in ClinicalTrials.gov was downloaded on September 27, 2010. At the time of the dataset download, 40,970 interventional trials had been registered since October 1, 2007. The osteoporosis subset comprised 239 interventional trials (0.6%). Those trials evaluating orthopedic procedures were excluded. The primary purpose was treatment in 67.0%, prevention in 20.1%, supportive care in 5.8%, diagnostic in 2.2%, basic science in 3.1%, health services research in 0.9%, and screening in 0.9%. The majority of studies (61.1%) included drug-related interventions. Most trials (56.9%) enrolled only women, 38.9% of trials were open to both men and women, and 4.2% enrolled only men. Roughly one fifth (19.7%) of trials excluded research participants older than 65 years, and 33.5% of trials excluded those older than 75 years. The funding sources were industry in 51.0%, the National Institutes of Health in 6.3%, and other in 42.7%. We found that most osteoporosis-related trials registered from October 2007 through September 2010 examined the efficacy and safety of drug treatment, and fewer trials examined prevention and non-drug interventions. Trials of interventions that are not required to be registered in ClinicalTrials.gov may be underrepresented. Few trials are specifically studying osteoporosis in men and older adults. Recently registered osteoporosis trials may not sufficiently address fracture prevention.

## Introduction

Osteoporosis is a common skeletal disorder characterized by decreased bone density, compromised bone strength, and increased fracture risk [1]. There were an estimated 9 million new osteoporotic fractures worldwide in 2000, and 1.6 million of these fractures occurred at the hip, representing a 25% increase in hip fractures since 1990. Hip fracture prevalence in men and women peaks between the ages of 75 and 79 years [2]. In 2005, the overall prevalence of osteoporosis or associated fracture among people enrolled in Medicare in the United States was 29.7% [3]. More than 1.5 million fractures occur annually in the United States and are associated with significant morbidity, mortality, and healthcare costs [4].

The burden of osteoporosis has been emphasized by the National Osteoporosis Foundation (NOF) [1] and the U.S. Department of Health and Human Services (HHS) [4]. The NOF has identified several areas that urgently require epidemiologic, clinical, and economic research: how to maximize peak bone mass in the young, the precise components of an effective exercise program for osteoporosis prevention and treatment, and duration of antiresorptive treatment [1]. The HHS has noted that osteoporosis is underdiagnosed and undertreated, and the agency has identified two goals in the area of osteoporosis in their Healthy People 2020 initiative: to reduce the proportion of adults with osteoporosis and to decrease the number of hip fractures among older adults [4].

To determine whether clinical trials are poised to address these issues, we examined recently registered osteoporosis trials in the ClinicalTrials.gov database. This manuscript is the result of the State of Clinical Trials project at the Clinical Trials Transformation Initiative [5].

## Materials and Methods

The methods used by ClinicalTrials.gov to register clinical trials have been described in detail elsewhere [6]. Briefly, trial sponsors and investigators from around the world can enter trial information through a web-based data entry system. The sample we examine in the present study includes trials registered to comply with statutory obligations, as well as those registered voluntarily to meet publication requirements or for other reasons. The Duke University Institutional Review Board granted exemption for this study, Pro00024659, on June 15, 2010.

### Creation of the ClinicalTrials.gov Dataset

A dataset of 96,346 clinical studies registered in ClinicalTrials.gov was downloaded in XML format on September 27, 2010. This download date was significant because it coincided with the anniversary of the enactment of the Food and Drug Administration Amendments Act 3 years earlier and the corresponding legal obligation for sponsors to register applicable interventional trials [7]. We next designed and implemented a relational database to facilitate aggregate analysis of data from ClinicalTrials.gov, as described in detail elsewhere [8].

### Creation of the Osteoporosis Study Dataset

**Fig 1:**
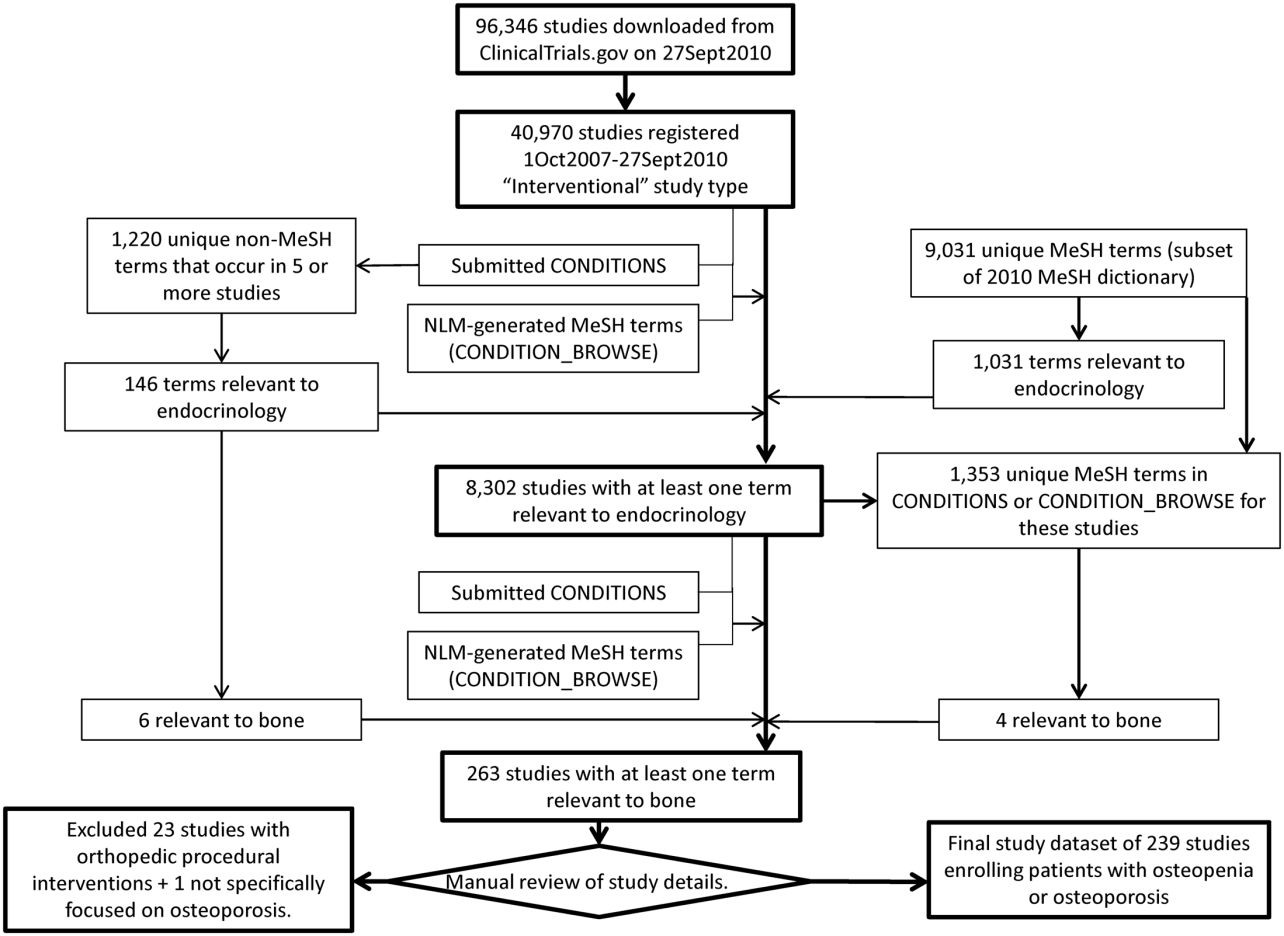
Flow Diagram Illustrating the Creation of the Osteoporosis Trials Dataset.

The analysis was restricted to the 40,970 trials classified as “interventional” study type registered between October 1, 2007, and September 27, 2010. (“Interventional” study type is defined by a ClinicalTrials.gov protocol element as “studies in human beings in which individuals are assigned by an investigator based on a protocol to receive specific interventions. Subjects may receive diagnostic, therapeutic or other types of interventions. The assignment of the intervention may or may not be random. The individuals are then followed and biomedical and/or health outcomes are assessed”—http://prsinfo.clinicaltrials.gov/definitions.html.) The osteoporosis study dataset (Fig 1) was created by using disease condition terms (Medical Subject Headings (MeSH) and non-MeSH) provided by the data submitters and additional condition MeSH terms generated by a National Library of Medicine (NLM) algorithm. A subset of the 2010 MeSH thesaurus from the NLM (http://www.nlm.nih.gov/mesh/filelist.html) and a list of non-MeSH disease condition terms provided by the data submitters that appeared in five or more studies in the selected analysis dataset were reviewed and annotated by clinical specialists in endocrinology, metabolism, and nutrition at Duke University School of Medicine (KB, WCL, BCB, JBG) and the University of Oxford (M. Angelyn Bethel, MD). As a first step, terms were annotated according to their relevance to the broad endocrinology domain. From a total of 9031 MeSH terms and 1220 non-MeSH terms reviewed, 1031 unique MeSH terms and 146 unique non-MeSH terms were annotated as relevant to the endocrinology domain. Of the 146 non-MeSH terms, six were relevant to bone (terms annotated were ‘osteopenia’, ‘postmenopausal osteoporosis’, ‘post-menopausal osteoporosis’, ‘hip fracture’, ‘low bone mineral density’, and ‘bone loss’). Using the endocrinology annotation, 8302 studies were identified with at least one condition term or condition MeSH term relevant to endocrinology. Restricted to these studies, 1353 unique MeSH terms occurred among the submitted conditions or NLM-generated MeSH terms. Of these, four were relevant to bone disease (terms annotated were ‘bone density’, ‘osteoporosis’, ‘osteoporosis, postmenopausal’, and ‘hip fractures’). Using the bone annotation, 263 studies were identified with at least one condition term or condition MeSH term relevant to bone. Trials with at least one relevant disease condition term were extracted and manually reviewed by one of the authors (KB). All studies with bone disease terms were included with the exception of one trial that was not specifically focused on osteoporosis and 23 pertaining to orthopedic procedural interventions. These trials were excluded because they focused on technical aspects of procedures such as orthopedic hardware (nails and screws) and intra-operative techniques. The final osteoporosis dataset comprised 239 trials.

### Derived Funding Source

The NLM defines the “lead sponsor” for a trial as the organization primarily responsible for study implementation and data analysis, and defines “collaborators” as those who provide other meaningful trial-related support including funding, design, implementation, data analysis, and reporting [6]. Agency names in these data elements are classified as industry, National Institutes of Health (NIH), U.S. federal government (excluding NIH), or other (manual review of the “other” category revealed that these were university or academic institutions). We derived probable funding source from the lead sponsor and collaborator fields using the following algorithm: If the lead sponsor was from industry, or if the NIH was neither a lead sponsor nor collaborator and at least one collaborator was from industry, then the study was categorized as industry-funded. If the lead sponsor was not from industry, and the NIH was either a lead sponsor or a collaborator, then the study was categorized as NIH-funded. Otherwise, if the lead sponsor and collaborator fields were non-missing, then the study was considered to be funded by other.

### Statistical Methods

Frequencies and percentages are provided for categorical trial characteristics. Missing values were excluded from denominators before calculating percentages. Medians and quartiles (Q1, Q3) are provided for continuous characteristics. For studies reporting an interventional model of “single group” and number of arms as “1,” the value of allocation (if missing) was assigned as “non-randomized” and the value of blinding (if missing) was assigned as “open.” Study duration was calculated from the study start date and the date when data collection on the primary endpoint was completed.

### Definition of Terms (from http://prsinfo.clinicaltrials.gov/definitions.html)

*Treatment*: protocol designed to evaluate one or more interventions for treating a disease, syndrome, or condition.

*Prevention*: protocol designed to assess one or more interventions aimed at preventing the development of a specific disease or health condition.

*Diagnostic*: protocol designed to evaluate one or more interventions aimed at identifying a disease or health condition.

*Supportive Care*: protocol designed to evaluate one or more interventions where the primary intent is to maximize comfort, minimize side effects, or mitigate against a decline in the subject’s health or function. In general, supportive care interventions are not intended to cure a disease.

*Screening*: protocol designed to assess or examine methods of identifying a condition (or risk factors for a condition) in people who are not yet known to have the condition (or risk factor).

*Health Services Research*: protocol designed to evaluate the delivery, processes, management, organization, or financing of healthcare.

*Basic Science*: protocol designed to examine the basic mechanism of action (e.g., physiology, biomechanics) of an intervention.

## Results

The detailed characteristics of the trials related to osteoporosis are outlined in S1–S6 Tables. Among the 40,970 interventional trials registered between October 1, 2007, and September 27, 2010, 239 (0.6%) were osteoporosis-related and had a mean duration of 2.3 years. Most trials (195/238, 81.9%) were performed in a randomized fashion, and 148/238 (62.1%) were double- or single-blinded (S1 Table). The majority of trials (184/231, 79.6%) had at least two arms, and 100/215 (46.5%) included an active comparator arm (S2 Table).

The primary purpose of the majority of trials (150/224, 67.0%) was treatment, whereas prevention was the primary purpose in 45/224 (20.1%) trials. Manual review of the latter trials by KB revealed that the primary preventive focus was drug therapy in 14, herbal and over-the-counter preparations in 11, exercise in 10, vitamin D and calcium supplementation in five, and nutrition in five. Two of the prevention trials evaluated bone mass accrual in healthy children and adolescents. For the remaining trials, the primary purposes were supportive care in 13/224 (5.8%), basic science in 7/224 (3.1%), diagnostic in 5/224 (2.2%), health services research in 2/224 (0.9%), and screening in 2/224 (0.9%), with 15/239 trials not reporting primary purpose (S3 Table).

In terms of intervention type, most studies (146/239, 61.1%) evaluated drug interventions. The primary outcomes listed for the drug intervention studies were as follows: bone density change by dual X-ray absorptiometry (63, 43%), safety and tolerability (19, 13%), bone turnover markers (13, 9%), calcium and vitamin D metabolism (8, 5%), bleeding or clotting (8, 5%), diagnostic radiological studies (MRI or peripheral quantitative computerized tomography) (7, 5%), urinary excretion bioequivalence, or pharmacokinetics (7, 5%), fractures (6, 4%), adherence (6, 4%), and patient satisfaction (6, 4%). One study determined survival, and two studies evaluated pain. Other study intervention types were dietary/supplement (36/239, 15.1%), behavioral (24/239, 10.0%), procedural (17/239, 7.1%), device (5/239, 2.1%), and biological (1/239, 0.4%) (S2 Table). Manual review of the latter revealed this intervention to be blood transfusion coming after hip fracture. Thirty-seven (15.5%) studies were categorized as “other” (evaluated a combination of the interventions). In terms of trial endpoints, the largest proportion of trials examined the endpoints of drug efficacy (83/206, 40.3%), safety (22/206, 10.7%), and both drug efficacy and safety (84/206, 40.8%). The other endpoints were bio-equivalence (5/206, 2.4%), pharmacokinetics (6/206, 2.9%), pharmacodynamics (3/206, 1.5%), and the combination of pharmacokinetics and pharmacodynamics (3/206, 1.5%) (S1 Table).

Although most trials (136/239, 56.9%) enrolled only women, 93/239 (38.9%) were open to both men and women, and 10/239 (4.2%) enrolled only men (S1 Table). When comparing the characteristics of trials restricting enrollment to men versus those restricting enrollment to women, the former were less likely to have a preventive purpose (1/9, 11.1% vs. 26/130, 20.0%) and more likely to focus on diagnosis (1/9, 11.1% vs. 2/130, 1.5%) and supportive care (2/9, 22.2% vs. 7/130, 5.4%). See S3 Table for detailed results.

In terms of age of trial participants, 210/239 (87.9%) studies targeted those age 18 years or older, whereas 8/239 (3.3%) targeted those age 18 years or younger (S1 and S3 Tables). When comparing the characteristics of trials with an age restriction versus those with no age restriction, trials targeting participants age 18 years or younger were more likely to cite prevention as their primary purpose (4/8, 50.0% vs. 41/216, 19.0%), enroll fewer participants (median enrollment 34.0 vs. 108.0), and be funded by a university or other academic institution (“other”) (6/8, 75.0% vs. 96/231, 41.6%) (S3 Table). Forty-seven (19.7%) trials excluded research participants older than 65 years, and 80/239 (33.5%) trials excluded those older than 75 years (S1 Table). Trials that excluded participants over the age of 65 years were more likely to have a preventive (14/43, 32.6% vs. 31/181, 17.1%) or basic science (5/43, 11.6% vs. 2/181, 1.1%) purpose in comparison with those trials without such an age exclusion (results not shown in table; available upon request).

The lead sponsor was industry in 105/239 (43.9%) trials, NIH in 3/239 (1.3%), U.S. government in 2/239 (0.8%), and “other” in 129/239 (54.0%) (S4 Table). Of the trials, 146/239 (61.1%) trials had only one sponsor or collaborator. Funding sources (incorporating information from both the lead sponsor and collaborator fields) were from industry in 122/239 (51.0%), NIH in 15/239 (6.3%), and “other” in 102/239 (42.7%). Data on number of centers per funding source were available for 209 studies. Among these, 41 (19.6%) were single-center industry-funded, 56 (26.8%) were multicenter industry-funded, 13 (6.2%) were single-center NIH-funded, 1 (0.5%) was multicenter NIH-funded, 84 (40.2%) were grouped as single-center funded by other, and the remaining 14 (6.7%) were multicenter other-sponsored trials.

A comparison of the characteristics of non-industry-funded and industry-funded trials (S5 Table) reveals the latter to be more treatment focused (82.6% vs. 50.5%) whereas non-industry-funded trials are focused more on prevention (28.4% vs. 12.2%), diagnosis (4.6% vs. 0%), supportive care (9.2% vs. 2.6%), health services research (1.8% vs. 0%), and basic science (4.6% vs. 1.7%). Furthermore, 88.5% of the trials funded by industry examined drug interventions compared to 32.5% of the non-industry-funded trials. In terms of study classification, the majority of industry- and non-industry-funded trials were classified as either safety or efficacy, or both. Industry-funded trials were more focused on safety (16.8% vs. 3.2%), whereas non-industry-funded trials were more focused on efficacy (64.5% vs. 20.4%).

Global distribution of trial facilities is represented graphically in S1 and S2 Figs. Of 209 trials with facility information, 85/209 (40.7%) reported trial facilities in the United States only, 19/209 (9.1%) reported facilities in both the United States and outside the country, and 105/209 (50.2%) had facilities only outside the country.

## Discussion

Our analysis of recently registered osteoporosis trials in the ClinicalTrials.gov database demonstrated that the majority of osteoporosis-related trials registered between October 2007 and September 2010 examined the efficacy and safety of drug treatment. Although drug intervention trials have the potential to support the charge of the HHS to decrease hip fractures in older adults [4], 20% of trials *excluded* research participants older than 65 years, and one in three trials *excluded* those older than 75 years. Those trials that *excluded* participants over 65 years of age were more likely to have a preventive focus. Therefore, the information obtained from these studies may not enhance our understanding of fracture prevention in these older, high-risk populations. This concern was highlighted recently by Järvinen et al., who noted that individuals older than 75 years are underrepresented or absent from most clinical trials examining the pharmacologic efficacy of hip fracture prevention [9]. Regardless of age exclusions, fewer trials in general examined prevention and non-drug interventions. Thus, the charge of the HHS to decrease the number of adults diagnosed with osteoporosis may not be fully addressed.

Reducing the proportion of adults with osteoporosis is critical because of the morbidity, mortality, and cost associated with osteoporotic fractures. With the rising prevalence of osteopenia and osteoporosis [1–11], associated fracture expenditures are projected to increase. In the European Union, the estimated direct cost of fracture treatment was €29 billion in 2010 [12,13]. Similarly, in the United States, the estimated direct medical cost of osteoporotic fractures in 2005 was $17 billion in 2005 [14]. These annual costs are expected to increase by 50% by 2025. Thus, studies evaluating fracture prevention through identification and treatment of at-risk populations are one way to reduce cost. However, in the ClinicalTrials.gov database, only 20% of trials cite prevention as their primary purpose. Furthermore, only a handful of the 45 prevention trials evaluated non-drug therapies such as specific forms of exercise, alternative therapies, or nutrition supplementation.

Investigation of bone health in younger individuals as well as gender and race subgroups has the potential to inform prevention and treatment strategies and lead to more generalizable recommendations. A small minority (3.3%) of all trials targeted subjects age 18 years or younger and aimed to enroll far fewer participants. Only two trials sought to address peak bone mass in healthy young subjects at a time when nutritional and exercise interventions may have an impact on peak bone mass.

Although only 29% of fractures occur in men [14], fracture-related mortality is higher in men than women [2]. Trombetti et al. documented mortality rates of 39% for men and 19% for women at 1 year after hip fracture [15]. This difference persisted after 7 years (85% vs. 67%). Almost 30% of fractures and a quarter of the total cost burden are borne by men [14]. Despite this significant disease burden in men, few trials in the ClinicalTrials.gov database are specifically studying osteoporosis in men. Those that are focused on male participants are less likely to have a preventive purpose.

There is a similar disparity regarding trials focused on racially diverse populations. The current prevalence of osteoporosis and fracture among the nonwhite population is lower than that in the white population [11]; however, the nonwhite population is predicted to bear an increasing share of the disease burden [14]. In fact, among Hispanic women living in California, hip fracture rates have doubled since 1983 [16]. There is no racial information data entry requirement in the ClinicalTrials.gov database; therefore we were unable to determine whether clinical trials registered during the time frame of interest will address treatment of osteoporosis among different racial groups.

Three additional gaps in the literature fuel the projected increased prevalence of disease and uncertainty regarding standard treatment regimens: 1) underdiagnosis and undertreatment of osteopenia/osteoporosis, 2) evaluation of the effect of combination or sequential therapy on fracture prevention, and 3) optimal duration of bisphosphonate treatment.

Despite known effective drug treatments and recommendations for therapy [17–27], many patients at risk for fracture are still underdiagnosed and undertreated [1,4]. The reasons for this are multifactorial and include healthcare provider, system, and patient barriers [28]. Less than 20% of patients with a fragility fracture receive treatment to prevent future fractures in the year after the fracture [29–31]. To better address this osteoporosis care gap, health services research trials are needed, but these types of trials made up only 0.9% of trials in the osteoporosis dataset.

Funding of trials contributes significantly to the types of trials conducted. Just over half of all the trials are funded by industry and may account for the relative lack of health services intervention and prevention trials in osteoporosis. It is perhaps not surprising that the majority of industry-funded trials are focused on treatment and drug interventions. The results of these trials have provided us with effective osteoporosis therapies in the clinical trial setting, but they do not address the unanswered questions of prevention and application to the growing community of older adults. It is encouraging that a relatively larger proportion of non-industry-funded trials are focused on prevention and health services research; however, these are the primary focus in only 21% of *all* clinical trials in the dataset.

While two-thirds of osteoporosis-related trials in the database cited their primary purpose as treatment, less than 50% reported an active comparator arm. One may argue that the “large number of untreated patients makes ‘no treatment’ a relevant comparator” [32]; however, given the costs of fractures and future predictions of a worsening burden, particularly among older adults, there is a need for expansion of clinical trials that compare effectiveness of pharmaceutical agents and evaluate combination or sequential therapy for fracture prevention.

In addition to defining better treatment algorithms, there is an urgent need for trials evaluating the optimal duration of osteoporosis treatment with bisphosphonates. This issue was identified by the NOF as an area for future research in their 2010 clinician’s guide to the treatment and prevention of osteoporosis [1]. Given the date of download of the ClinicalTrials.gov dataset and the median trial duration of 2 years, the current clinical trials dataset is not poised to answer the question of long-term efficacy and safety of pharmacologic agents such as bisphosphonates.

Global distribution of trials reveals a paucity of studies with participating facilities on the African continent and in the developing world where data on the incidence of osteoporotic fractures is sparse. In these economically disadvantaged countries, trials are needed to determine which osteoporosis therapies can be utilized in a practical and cost-effective fashion.

There are several limitations to drawing conclusions from the ClinicalTrials.gov database, and these have been previously outlined [33,34]. Limitations pertinent to the osteoporosis-related trials include the following. First, while ClinicalTrials.gov encompasses the majority (80%) of trials in the World Health Organization portal, it is not a comprehensive database of clinical trials worldwide. This is particularly important for the osteoporosis dataset where we have emphasized the need for health services research trials to address the osteoporosis care gap. Those health services research trials that do not involve a drug, biological, or device may not be registered within the ClinicalTrials.gov database and thus are not included in this analysis. Second, our data collection and analysis depended on accurate entry of the data into the various fields on the ClinicalTrials.gov website. Since the requirements and methods for data entry have changed over time, there may be inaccuracies and variability within the data collected. In addition, fields with data missing or categorized as “other” posed challenges for analysis, especially in the lead sponsor/collaborator fields. Third, we cannot correlate trial activity within a particular geographic area with population density of that area because we do not have information on the number of unique trial sites per country. Finally, the current study is the result of a single overview of the dataset, and we could not examine whether trial characteristics had changed over time.

This analysis suggests that recently registered osteoporosis trials may not sufficiently address osteoporosis prevention and treatment in at-risk populations. Although our dataset may not reflect all relevant ongoing clinical trials in osteoporosis, it does provide a relatively comprehensive overview of recent interventional trials and is the only review that has been published on the subject. This information may be useful in the identification of issues requiring further study.

## Supporting Information

S1 Fig U.S. States Where Osteoporosis Studies Have Facilities. (TIF)

S2 Fig Countries Where Osteoporosis Studies Have Facilities. (TIFF)

S1 Table Characteristics of Osteoporosis-Related Interventional Trials Registered With ClinicalTrials.gov, 2007–2010. (DOCX)

S2 Table Study Arms and Intervention Types of Osteoporosis Studies. (DOCX)

S3 Table Selected Characteristics of Bone Studies by Gender Restrictions on Enrollment and by Whether Enrollment Was Restricted to Children. (DOCX)

S4 Table Sponsors and Collaborators of Osteoporosis Studies. (DOCX)

S5 Table Selected Characteristics of Bone Studies by Funding Source. (DOCX)

S6 Table Location of Facilities for Osteoporosis Studies. (DOCX)
