# Supplementary figures and images for: Recent Clinical Trials in Osteoporosis: A Firm Foundation or Falling Short?
Source: PLoS One. 2016 May 18;11(5):e0156068. doi: 10.1371/journal.pone.0156068 (PMC4871563; doi:10.1371/journal.pone.0156068)

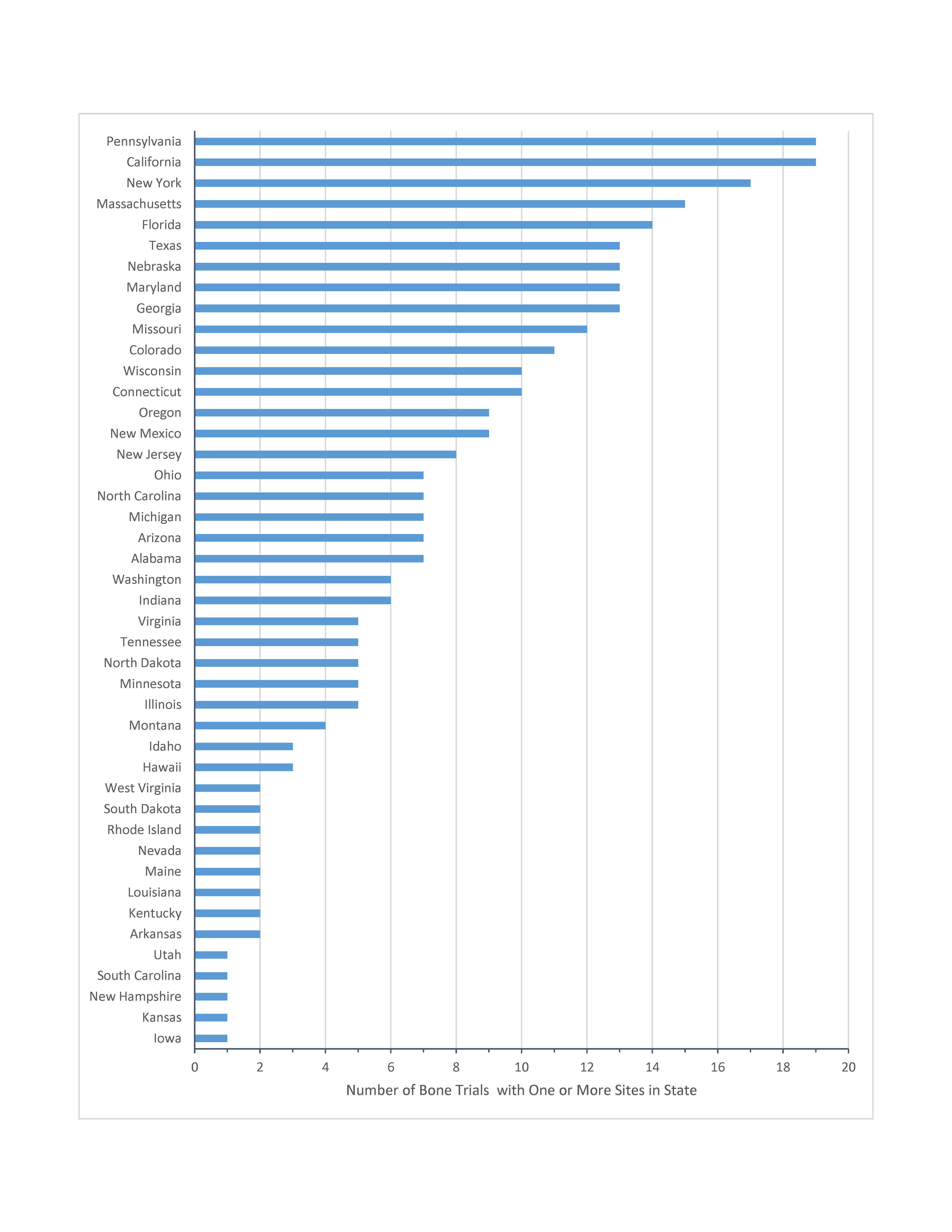

Supplement: S1 Fig — (TIF) [file pone.0156068.s001.tif]

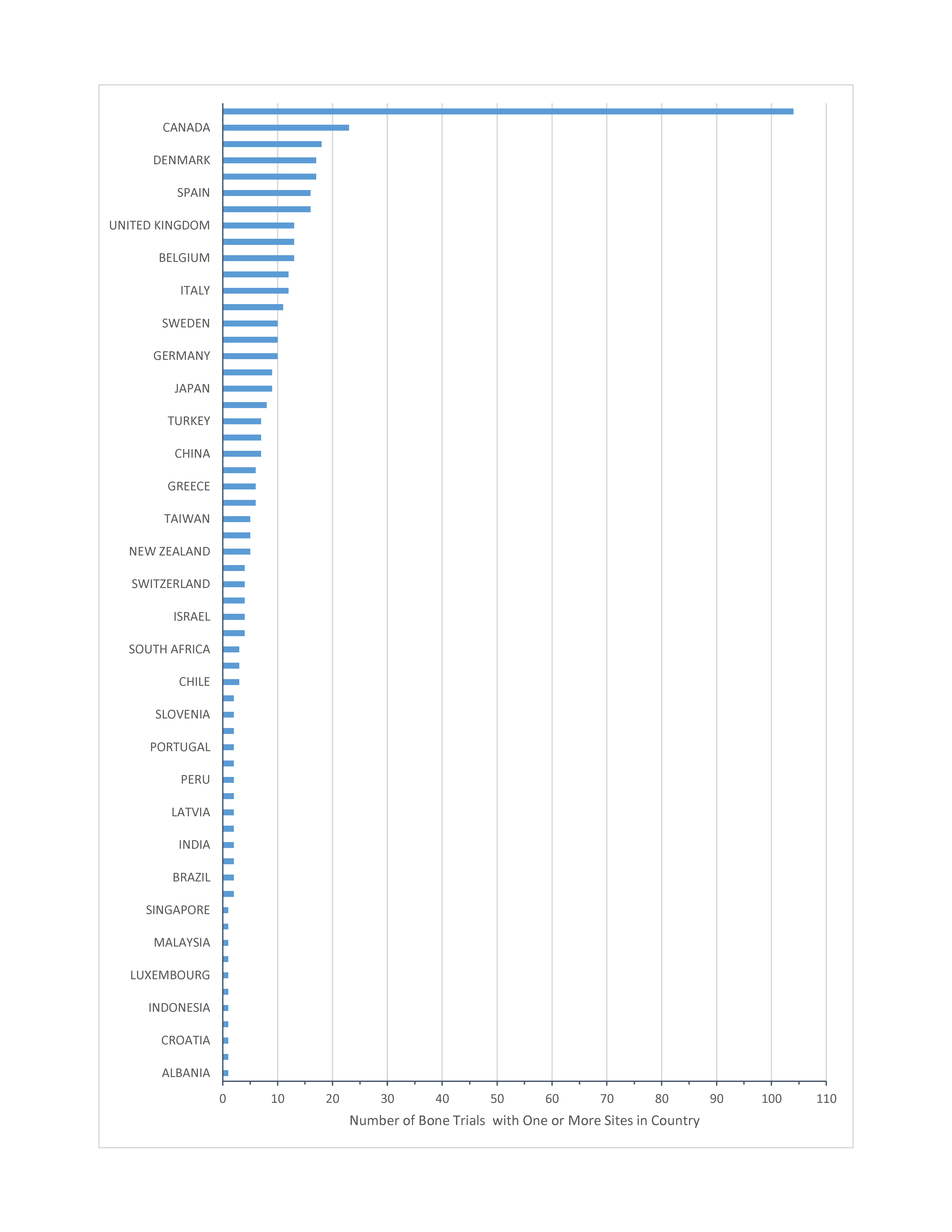

Supplement: S2 Fig — (TIFF) [file pone.0156068.s002.tiff]
