# Supplementary material for: Recent Clinical Trials in Osteoporosis: A Firm Foundation or Falling Short?
Source: PLoS One. 2016 May 18;11(5):e0156068. doi: 10.1371/journal.pone.0156068 (PMC4871563; doi:10.1371/journal.pone.0156068)
Supplement: S1 Table — (DOCX) [file pone.0156068.s003.docx]

| **Characteristic** | **Osteoporosis studies (N=239)^a^** |
| --- | --- |
| **Study start year** |  |
| Before 2007 | 74/230 (32.2) |
| 2007 | 21/230 (9.1) |
| 2008 | 60/230 (26.1) |
| 2009 | 45/230 (19.6) |
| 2010 or later | 30/230 (13.0) |
| Start year missing | 9/239 (3.8) |
| **Study completion year** |  |
| <2007 | 29/210 (13.8) |
| 2007 | 11/210 (5.2) |
| 2008 | 20/210 (9.5) |
| 2009 | 38/210 (18.1) |
| 2010 | 39/210 (18.6) |
| 2011 | 37/210 (17.6) |
| >2011 | 36/210 (17.1) |
| Completion year missing | 29/239 (12.1) |
| **Completion type** |  |
| Actual | 90/210 (42.9) |
| Anticipated | 120/210 (57.1) |
| Completion type missing | 29/239 (12.1) |
| **Overall status** |  |
| Not yet recruiting | 20/239 (8.4) |
| Recruiting | 55/239 (23.0) |
| Enrolling by invitation | 4/239 (1.7) |
| Active, not recruiting | 53/239 (22.2) |
| Completed | 98/239 (41.0) |
| Suspended | 2/239 (0.8) |
| Terminated | 5/239 (2.1) |
| Withdrawn | 2/239 (0.8) |
| Overall status missing | 0 |
| **Primary completion year^b^** |  |
| <2007 | 24/205 (11.7) |
| 2007 | 11/205 (5.4) |
| 2008 | 20/205 (9.8) |
| 2009 | 40/205 (19.5) |
| 2010 | 42/205 (20.5) |
| 2011 | 38/205 (18.5) |
| >2011 | 30/205 (14.6) |
| Primary completion year missing | 34/239 (14.2) |
| **Primary completion type** |  |
| Actual | 80/205 (39.0) |
| Anticipated | 125/205 (61.0) |
| Primary completion type missing | 34/239 (14.2) |
| **Years to primary completion,** **N** | 205 |
| Mean ± SD | 2.3 ± 2.31 |
| Median | 2 |
| Q1, Q3 | 1.0, 3.1 |
| Min, max | 0.0, 25.2 |
| Years to primary completion missing | 34/239 (14.2) |
| **Primary purpose** |  |
| Treatment | 150/224 (67.0) |
| Prevention | 45/224 (20.1) |
| Supportive care | 13/224 (5.8) |
| Basic science | 7/224 (3.1) |
| Diagnostic | 5/224 (2.2) |
| Screening | 2/224 (0.9) |
| Health services research | 2/224 (0.9) |
| Primary purpose missing | 15/239 (6.3) |
| **Study classification** |  |
| Safety/efficacy | 84/206 (40.8) |
| Efficacy | 83/206 (40.3) |
| Safety | 22/206 (10.7) |
| Bio-equivalence | 5/206 (2.4) |
| Pharmacokinetics | 6/206 (2.9) |
| Pharmacodynamics | 3/206 (1.5) |
| Pharmacokinetics/dynamics | 3/206 (1.5) |
| Study classification missing | 33/239 (13.8) |
| **Interventional model** |  |
| Single group | 54/238 (22.7) |
| Parallel | 162/238 (68.1) |
| Crossover | 15/238 (6.3) |
| Factorial | 7/238 (2.9) |
| Intervention model missing | 1/239 (0.4) |
| **Masking** |  |
| Open | 90/238 (37.8) |
| Single blind | 42/238 (17.6) |
| Double blind | 106/238 (44.5) |
| Masking missing | 1/239 (0.4) |
| **Allocation** |  |
| Randomized | 195/238 (81.9) |
| Nonrandomized | 43/238 (18.1) |
| Allocation missing | 1/239 (0.4) |
| **Phase** |  |
| Phase 0 | 2/239 (0.8) |
| Phase 1 | 27/239 (11.3) |
| Phase 1/Phase 2 | 4/239 (1.7) |
| Phase 2 | 37/239 (15.5) |
| Phase 2/Phase 3 | 3/239 (1.3) |
| Phase 3 | 53/239 (22.2) |
| Phase 4 | 47/239 (19.7) |
| N/A | 66/239 (27.6) |
| Phase missing | 0 |
| **Enrollment** |  |
| 1 to 10 | 8/239 (3.3) |
| 11 to 50 | 56/239 (23.4) |
| 51 to 100 | 54/239 (22.6) |
| 101 to 500 | 85/239 (35.6) |
| 501 to 1,000 | 22/239 (9.2) |
| 1,001 to 2,000 | 4/239 (1.7) |
| 2,000 to 5,000 | 8/239 (3.3) |
| 5,000 to 10,000 | 1/239 (0.4) |
| More than 10,000 | 1/239 (0.4) |
| Enrollment missing | 0 |
| **Enrollment type** |  |
| Actual | 109/238 (45.8) |
| Anticipated | 129/238 (54.2) |
| Enrollment type missing | 1/239 (0.4) |
| **Gender** |  |
| Female | 136/239 (56.9) |
| Male | 10/239 (4.2) |
| Both | 93/239 (38.9) |
| Gender missing | 0 |
| **Accepts healthy volunteers** | 70/237 (29.5) |
| Healthy volunteers missing | 2/239 (0.8) |
| **Minimum age (years)** |  |
| Under 5 | 2/239 (0.8) |
| 5 to 17 | 9/239 (3.8) |
| 18 to 21 | 56/239 (23.4) |
| 22 to 49 | 58/239 (24.3) |
| 50 or more | 96/239 (40.2) |
| NA | 18/239 (7.5) |
| Minimum age missing | 0 |
| **Maximum age (years)** |  |
| 5 to 17 | 5/239 (2.1) |
| 18 to 49 | 16/239 (6.7) |
| 50 to 59 | 8/239 (3.3) |
| 60 to 69 | 19/239 (7.9) |
| 70 to 79 | 32/239 (13.4) |
| 80 or more | 54/239 (22.6) |
| NA | 105/239 (43.9) |
| Maximum age missing | 0 |
| Study has maximum age of ≤18 | 8/239 (3.3) |
| Study has minimum age of ≥65 | 25/239 (10.5) |
| Study excludes ages >65 | 47/239 (19.7) |
| Study has minimum age of ≥75 | 1/239 (0.4) |
| Study excludes ages >75 | 80/239 (33.5) |
| **Has DMC** | 70/189 (37.0) |
| **Has DMC missing** | 50/239 (20.9) |
| **Results received** | 7/239 (2.9) |

Values are given as numerator/denominator (%), except where otherwise noted.

^a^Missing values are excluded from denominators before calculating percentages.

^b^Derived: years from start date to completion of follow-up for primary endpoint.
