# Supplementary material for: Recent Clinical Trials in Osteoporosis: A Firm Foundation or Falling Short?
Source: PLoS One. 2016 May 18;11(5):e0156068. doi: 10.1371/journal.pone.0156068 (PMC4871563; doi:10.1371/journal.pone.0156068)
Supplement: S2 Table — (DOCX) [file pone.0156068.s004.docx]

| **Characteristic** | **Osteoporosis studies (N=239)^a^** |
| --- | --- |
| **Number of arms** |  |
| One | 47/231 (20.3) |
| Two | 126/231 (54.5) |
| Three | 28/231 (12.1) |
| Four | 21/231 (9.1) |
| Five or more | 9/231 (3.9) |
| **Arm types^b^** |  |
| Active comparator | 100/215 (46.5) |
| No intervention arm | 33/215 (15.3) |
| Experimental arm | 159/215 (74.0) |
| Placebo comparator arm | 79/215 (36.7) |
| Sham comparator arm | 3/215 (1.4) |
| Other arm | 11/215 (5.1) |
| **Intervention types^c^** |  |
| Drug intervention | 146/239 (61.1) |
| “Other” intervention | 37/239 (15.5) |
| Dietary supplement intervention | 36/239 (15.1) |
| Behavioral intervention | 24/239 (10.0) |
| Procedure intervention | 17/239 (7.1) |
| Device intervention | 5/239 (2.1) |
| Biological intervention | 1/239 (0.4) |
| Radiation intervention | 1/239 (0.4) |
| Genetic intervention | 0 |

Values are given as numerator/denominator (%).

^a^Missing values are excluded from denominators before calculating percentages.

^b^A study may have several arm types and may be counted in more than one row.

^c^A study may have several intervention types and may be counted in more than one row.
