# Supplementary material for: Recent Clinical Trials in Osteoporosis: A Firm Foundation or Falling Short?
Source: PLoS One. 2016 May 18;11(5):e0156068. doi: 10.1371/journal.pone.0156068 (PMC4871563; doi:10.1371/journal.pone.0156068)
Supplement: S3 Table — (DOCX) [file pone.0156068.s005.docx]

| **Characteristic** | **Male (N=10)** | **Female (N=136)** | **Both (N=93)** | **Restricted to age ≤18 years (N=8)** | **Not restricted (N=231)** |
| --- | --- | --- | --- | --- | --- |
| **Primary purpose** |  |  |  |  |  |
| Treatment | 5/9 (55.6) | 90/130 (69.2) | 55/85 (64.7) | 4/8 (50.0) | 146/216 (67.6) |
| Prevention | 1/9 (11.1) | 26/130 (20.0) | 18/85 (21.2) | 4/8 (50.0) | 41/216 (19.0) |
| Diagnostic | 1/9 (11.1) | 2/130 (1.5) | 2/85 (2.4) | 0/8 (0.0) | 5/216 (2.3) |
| Supportive Care | 2/9 (22.2) | 7/130 (5.4) | 4/85 (4.7) | 0/8 (0.0) | 13/216 (6.0) |
| Screening | 0/9 (0.0) | 2/130 (1.5) | 0/85 (0.0) | 0/8 (0.0) | 2/216 (0.9) |
| Health Services Research | 0/9 (0.0) | 0/130 (0.0) | 2/85 (2.4) | 0/8 (0.0) | 2/216 (0.9) |
| Basic Science | 0/9 (0.0) | 3/130 (2.3) | 4/85 (4.7) | 0/8 (0.0) | 7/216 (3.2) |
| **Study classification** |  |  |  |  |  |
| Safety | 2/8 (25.0) | 10/119 (8.4) | 10/79 (12.7) | 0/7 (0.0) | 22/199 (11.1) |
| Efficacy | 3/8 (37.5) | 48/119 (40.3) | 32/79 (40.5) | 4/7 (57.1) | 79/199 (39.7) |
| Safety/Efficacy | 3/8 (37.5) | 52/119 (43.7) | 29/79 (36.7) | 3/7 (42.9) | 81/199 (40.7) |
| Bio-equivalence | 0/8 (0.0) | 1/119 (0.8) | 4/79 (5.1) | 0/7 (0.0) | 5/199 (2.5) |
| Pharmacokinetics | 0/8 (0.0) | 3/119 (2.5) | 3/79 (3.8) | 0/7 (0.0) | 6/199 (3.0) |
| Pharmacodynamics | 0/8 (0.0) | 3/119 (2.5) | 0/79 (0.0) | 0/7 (0.0) | 3/199 (1.5) |
| Pharmacokinetics/Dynamics | 0/8 (0.0) | 2/119 (1.7) | 1/79 (1.3) | 0/7 (0.0) | 3/199 (1.5) |
| **Phase** |  |  |  |  |  |
| Phase 0 | 0/10 (0.0) | 1/136 (0.7) | 1/93 (1.1) | 0/8 (0.0) | 2/231 (0.9) |
| Phase 1 | 1/10 (10.0) | 13/136 (9.6) | 13/93 (14.0) | 0/8 (0.0) | 27/231 (11.7) |
| Phase 1/Phase 2 | 0/10 (0.0) | 2/136 (1.5) | 2/93 (2.2) | 1/8 (12.5) | 3/231 (1.3) |
| Phase 2 | 0/10 (0.0) | 28/136 (20.6) | 9/93 (9.7) | 0/8 (0.0) | 37/231 (16.0) |
| Phase 2/Phase 3 | 0/10 (0.0) | 2/136 (1.5) | 1/93 (1.1) | 0/8 (0.0) | 3/231 (1.3) |
| Phase 3 | 5/10 (50.0) | 24/136 (17.6) | 24/93 (25.8) | 4/8 (50.0) | 49/231 (21.2) |
| Phase 4 | 1/10 (10.0) | 33/136 (24.3) | 13/93 (14.0) | 0/8 (0.0) | 47/231 (20.3) |
| N/A | 3/10 (30.0) | 33/136 (24.3) | 30/93 (32.3) | 3/8 (37.5) | 63/231 (27.3) |
| **Enrollment** |  |  |  |  |  |
| 1 to 10 | 1/10 (10.0) | 4/136 (2.9) | 3/93 (3.2) | 0/8 (0.0) | 8/231 (3.5) |
| 11 to 50 | 2/10 (20.0) | 33/136 (24.3) | 21/93 (22.6) | 5/8 (62.5) | 51/231 (22.1) |
| 51 to 100 | 3/10 (30.0) | 28/136 (20.6) | 23/93 (24.7) | 2/8 (25.0) | 52/231 (22.5) |
| 101 to 500 | 3/10 (30.0) | 47/136 (34.6) | 35/93 (37.6) | 1/8 (12.5) | 84/231 (36.4) |
| 501 to 1,000 | 1/10 (10.0) | 15/136 (11.0) | 6/93 (6.5) | 0/8 (0.0) | 22/231 (9.5) |
| 1,001 to 2,000 | 0/10 (0.0) | 2/136 (1.5) | 2/93 (2.2) | 0/8 (0.0) | 4/231 (1.7) |
| 2,000 to 5,000 | 0/10 (0.0) | 5/136 (3.7) | 3/93 (3.2) | 0/8 (0.0) | 8/231 (3.5) |
| 5,000 to 10,000 | 0/10 (0.0) | 1/136 (0.7) | 0/93 (0.0) | 0/8 (0.0) | 1/231 (0.4) |
| More than 10,000 | 0/10 (0.0) | 1/136 (0.7) | 0/93 (0.0) | 0/8 (0.0) | 1/231 (0.4) |
| **Enrollment, n** | 10 | 136 | 93 | 8 | 231 |
| Median (Q1, Q3) | 95.0 (40.0, 232.0) | 112.5 (46.5, 249.5) | 100.0 (50.0, 273.0) | 34.0 (27.0, 73.5) | 108.0 (50.0, 273.0) |
| **Funding source^a^** |  |  |  |  |  |
| Industry | 6/10 (60.0) | 78/136 (57.4) | 38/93 (40.9) | 2/8 (25.0) | 120/231 (51.9) |
| NIH | 0/10 (0.0) | 7/136 (5.1) | 8/93 (8.6) | 0/8 (0.0) | 15/231 (6.5) |
| Other | 4/10 (40.0) | 51/136 (37.5) | 47/93 (50.5) | 6/8 (75.0) | 96/231 (41.6) |
| **Lead sponsor** |  |  |  |  |  |
| Industry | 5/10 (50.0) | 69/136 (50.7) | 31/93 (33.3) | 1/8 (12.5) | 104/231 (45.0) |
| NIH | 0/10 (0.0) | 1/136 (0.7) | 2/93 (2.2) | 0/8 (0.0) | 3/231 (1.3) |
| U.S. federal government | 0/10 (0.0) | 2/136 (1.5) | 0/93 (0.0) | 0/8 (0.0) | 2/231 (0.9) |
| Other | 5/10 (50.0) | 64/136 (47.1) | 60/93 (64.5) | 7/8 (87.5) | 122/231 (52.8) |
| **Study has one or more of these intervention types** |  |  |  |  |  |
| Drug | 7/10 (70.0) | 85/136 (62.5) | 54/93 (58.1) | 5/8 (62.5) | 141/231 (61.0) |
| Procedure | 1/10 (10.0) | 7/136 (5.1) | 9/93 (9.7) | 0/8 (0.0) | 17/231 (7.4) |
| Biological/vaccine | 0/10 (0.0) | 0/136 (0.0) | 1/93 (1.1) | 0/8 (0.0) | 1/231 (0.4) |
| Behavioral | 2/10 (20.0) | 14/136 (10.3) | 8/93 (8.6) | 0/8 (0.0) | 24/231 (10.4) |
| Device | 0/10 (0.0) | 1/136 (0.7) | 4/93 (4.3) | 0/8 (0.0) | 5/231 (2.2) |
| Radiation | 0/10 (0.0) | 1/136 (0.7) | 0/93 (0.0) | 0/8 (0.0) | 1/231 (0.4) |
| Dietary supplement | 1/10 (10.0) | 25/136 (18.4) | 10/93 (10.8) | 3/8 (37.5) | 33/231 (14.3) |
| Genetic | 0/10 (0.0) | 0/136 (0.0) | 0/93 (0.0) | 0/8 (0.0) | 0/231 (0.0) |
| Other | 2/10 (20.0) | 16/136 (11.8) | 19/93 (20.4) | 4/8 (50.0) | 33/231 (14.3) |

Values are given as numerator/denominator (%), except where otherwise noted.

^a^Derived from lead sponsor and collaborator fields using the “Derived funding source” algorithm described in Methods.
