# Supplementary material for: Recent Clinical Trials in Osteoporosis: A Firm Foundation or Falling Short?
Source: PLoS One. 2016 May 18;11(5):e0156068. doi: 10.1371/journal.pone.0156068 (PMC4871563; doi:10.1371/journal.pone.0156068)
Supplement: S4 Table — (DOCX) [file pone.0156068.s006.docx]

| **Characteristic** | **Osteoporosis studies (N=239)^a^** |
| --- | --- |
| **Lead sponsor classification^b^** |  |
| Industry | 105/239 (43.9) |
| NIH | 3/239 (1.3) |
| U.S. federal government | 2/239 (0.8) |
| Other | 129/239 (54.0) |
| **One sponsor or collaborator** | 146/239 (61.1) |
| **Collaborators^c^** |  |
| Industry collaborator | 32/239 (13.4) |
| NIH collaborator | 12/239 (5.0) |
| U.S. federal gov’t collaborator | 1/239 (0.4) |
| Other collaborator | 53/239 (22.2) |
| **Lead sponsor or collaborators** |  |
| Industry lead or collaborator | 122/239 (51.0) |
| NIH lead or collaborator | 15/239 (6.3) |
| U.S. federal gov’t lead or collaborator | 3/239 (1.3) |
| Other lead or collaborator | 134/239 (56.1) |
| **Funding source^d^** |  |
| Industry | 122/239 (51.0) |
| NIH | 15/239 (6.3) |
| Other | 102/239 (42.7) |
| **Funding source/number of centers classification** |  |
| Industry-funded single-center | 41/209 (19.6) |
| Industry-funded multi-center | 56/209 (26.8) |
| NIH-funded single-center | 13/209 (6.2) |
| NIH-funded multicenter | 1/209 (0.5) |
| Other-single-center | 84/209 (40.2) |
| Other-multicenter | 14/209 (6.7) |

Values are given as numerator/denominator (%).

^a^Missing values are excluded from denominators before calculating percentages.

^b^Each study has one lead sponsor.

^c^A study may have several collaborators.

^d^Derived from lead sponsor and collaborator fields using the “Derived funding source” algorithm described in Methods.
