# Supplementary material for: Recent Clinical Trials in Osteoporosis: A Firm Foundation or Falling Short?
Source: PLoS One. 2016 May 18;11(5):e0156068. doi: 10.1371/journal.pone.0156068 (PMC4871563; doi:10.1371/journal.pone.0156068)
Supplement: S5 Table — (DOCX) [file pone.0156068.s007.docx]

| **Characteristic** | **All bone (N=239)** | **Industry (N=122)** | **Non-industry (N=117)** |
| --- | --- | --- | --- |
| **Primary purpose** |  |  |  |
| Treatment | 150/224 (67.0) | 95/115 (82.6) | 55/109 (50.5) |
| Prevention | 45/224 (20.1) | 14/115 (12.2) | 31/109 (28.4) |
| Diagnostic | 5/224 (2.2) | 0/115 (0.0) | 5/109 (4.6) |
| Supportive care | 13/224 (5.8) | 3/115 (2.6) | 10/109 (9.2) |
| Screening | 2/224 (0.9) | 1/115 (0.9) | 1/109 (0.9) |
| Health services research | 2/224 (0.9) | 0/115 (0.0) | 2/109 (1.8) |
| Basic science | 7/224 (3.1) | 2/115 (1.7) | 5/109 (4.6) |
| **Study has one or more of these intervention types** |  |  |  |
| Drug | 146/239 (61.1) | 108/122 (88.5) | 38/117 (32.5) |
| Procedure | 17/239 (7.1) | 2/122 (1.6) | 15/117 (12.8) |
| Biological/vaccine | 1/239 (0.4) | 0/122 (0.0) | 1/117 (0.9) |
| Behavioral | 24/239 (10.0) | 1/122 (0.8) | 23/117 (19.7) |
| Device | 5/239 (2.1) | 1/122 (0.8) | 4/117 (3.4) |
| Radiation | 1/239 (0.4) | 1/122 (0.8) | 0/117 (0.0) |
| Dietary supplement | 36/239 (15.1) | 14/122 (11.5) | 22/117 (18.8) |
| Genetic | 0/239 (0.0) | 0/122 (0.0) | 0/117 (0.0) |
| Other | 37/239 (15.5) | 8/122 (6.6) | 29/117 (24.8) |
| **Study classification** |  |  |  |
| Safety | 22/206 (10.7) | 19/113 (16.8) | 3/93 (3.2) |
| Efficacy | 83/206 (40.3) | 23/113 (20.4) | 60/93 (64.5) |
| Safety/efficacy | 84/206 (40.8) | 56/113 (49.6) | 28/93 (30.1) |
| Bio-equivalence | 5/206 (2.4) | 4/113 (3.5) | 1/93 (1.1) |
| Pharmacokinetics | 6/206 (2.9) | 5/113 (4.4) | 1/93 (1.1) |
| Pharmacodynamics | 3/206 (1.5) | 3/113 (2.7) | 0/93 (0.0) |
| Pharmacokinetics/dynamics | 3/206 (1.5) | 3/113 (2.7) | 0/93 (0.0) |

Values are given as numerator/denominator (%).
