# Supplementary material for: Recent Clinical Trials in Osteoporosis: A Firm Foundation or Falling Short?
Source: PLoS One. 2016 May 18;11(5):e0156068. doi: 10.1371/journal.pone.0156068 (PMC4871563; doi:10.1371/journal.pone.0156068)
Supplement: S6 Table — (DOCX) [file pone.0156068.s008.docx]

| **Characteristic** | **Osteoporosis studies (N=239)^a^** |
| --- | --- |
| **Number of facilities, N** | 209 |
| Mean ± SD | 7.8 ± 21.95 |
| Median | 1 |
| Q1, Q3 | 1.0, 3.0 |
| Min, max | 1.0, 191.0 |
| **Number of facilities** |  |
| Single facility | 138/209 (66.0) |
| Multiple facilities | 71/209 (34.0) |
| **Regions where studies have facilities^b^** |  |
| Africa | 3/209 (1.4) |
| Central America | 0 |
| Eastern Asia | 23/209 (11.0) |
| Europe | 76/209 (36.4) |
| Middle East | 12/209 (5.7) |
| North America | 117/209 (56.0) |
| North Asia | 6/209 (2.9) |
| Pacifica | 17/209 (8.1) |
| South America | 13/209 (6.2) |
| South Asia | 2/209 (1.0) |
| Southeast Asia | 7/209 (3.3) |
| Region missing | 30/239 (12.6) |
| **Has an enrolling facility in United States** | 104/209 (49.8) |
| **Location of facilities relative to United States** |  |
| Facilities in United States only | 85/209 (40.7) |
| Facilities in United States and rest of world | 19/209 (9.1) |
| Facilities in rest of world only | 105/209 (50.2) |

Values are given as numerator/denominator (%), except where otherwise noted.

^a^Missing values are excluded from denominators before calculating percentages.

^b^A study may have several facilities in one or more regions and may be counted in more than one row. Regions defined as at http://www.clinicaltrials.gov/ct2/search/browse?brwse=locn_cat.
